# Qualitative indicators of milk of Simmental and Holstein cows in different seasons of lactation

**DOI:** 10.14202/vetworld.2021.956-963

**Published:** 2021-04-21

**Authors:** Aigerim Zhanuzakovna Khastayeva, Venera Serikbayevna Zhamurova, Laura Asilbekovna Mamayeva, Akylzhan Turalievish Kozhabergenov, Nurlybek Zhanybekovich Karimov, Karlygash Muratbekkyzy Muratbekova

**Affiliations:** 1 Department of Technology and Food Safety, Kazakh National Agrarian University, Abai Avenue, 8, Almaty 050010, Kazakhstan; 2 Testing Center, LLP Kazakh Scientific Research Institute of Livestock and Fodder Production, Zhandosov Street, 51, Almaty 050035, Kazakhstan

**Keywords:** cow’s milk, fatty acid composition, Holstein, season, Simmental breed

## Abstract

**Background and Aim:**

Milk producers need to ensure that their cows are producing high-quality, nutritional milk, which is influenced by the breed, age, nutrition, and health status of lactating animals. The aim of this study was to determine the effect of animal breed, season, and milk production on the physicochemical characteristics of milk and predicting the effect of these changes on the composition and quality of milk products.

**Materials and Methods:**

In total, 80 milk samples (40 Simmental and 40 Holstein) were analyzed from LLP “Kirova” of Pavlodar region (Simmental breed) and JSC “Astana-Onim” of Akmola region (Holstein breed) in the Republic of Kazakhstan. The physicochemical parameters, including fatty acid (FA) content, were studied.

**Results:**

The Simmental cows had the lowest mass fraction of fat in the spring at 3.94% and the highest fat content in the winter at 4.09%, which was the overall highest fat content measured in both breeds (p<0.001). The lowest protein in the Simmentals was also measured in the spring. The Holstein cows had the highest fat content at 3.8% and the highest protein content at 3.3% in autumn.

**Conclusion:**

It was found that the season and breed significantly affected the physicochemical parameters and the FA contents in cow milk. The superiority of the Simmental cows over the Holstein cows throughout the year was statistically significant.

## Introduction

Milk is a biological fluid with a high nutritional value. Its constituents include, for example, water, milk fat, proteins, lactose, and minerals. Since the quality of dairy products depends largely on the composition of raw bulk milk, factors responsible for changes to the composition and physicochemical properties of raw milk are of paramount importance. The main factors influencing the composition of milk are season, lactation stage, feeding, milking interval, breed, and age of the dairy cow [1]. The effect of seasonal fluctuations on milk yield (MY) and milk composition has been widely researched [1-3]. Milk components vary based on milking time, DIM, season age, and health of the cow [4,5].

Milk protein is an important indicator of milk quality. The protein content in milk reflects whether the cow is supplied with energy and is an energy barometer for the herd. Specifically, the amount of protein in milk depends on whether enough energy is available to the rumen microbes that synthesize microbial protein [6]. Milk fat is a complex consisting of simple lipids (triglycerides, diglycerides, and monoglycerides), complex lipids (phospholipids, lecithin, cephalin, and sphingomyelin), derivatives of lipids (free fatty acids [FA]), and substances associated with fat (sterols, cholesterol, fat-soluble Vitamins A, E, D, and K, and carotenoids) [7].

The secretion of milk fat and the composition of lactic FA are of great interest to human nutrition; their modification in dairy cows through dietary manipulations has attracted considerable research attention [8]. Milk fat contains more than 400 FAs, which occur partly from the synthesis of the breast (almost 50%), partly from the diet, which is affected by the process of rubenbiohydrogenation, and due to the immobilization of animal fat deposits [9]. Some FA classes, such as branched-chain FA and cis-and trans-isomers 18:1, 18:2, and 18:3, are associated with rumen activity [10-12], while other FA classes, such as *de novo* FA, and classes with 18 carbon chains are also associated with energy exchange [12-14]. Thus, since the FA profile of milk can be viewed as a trace of the cow’s nutrition and metabolism, an analysis of MY variability and the FA profile can be used to draw conclusions regarding various farming or forage systems, herds, or even factors that influence the health status of the cows.

Milk quality is also assessed using sanitary and hygienic indicators that can be used to judge the freshness of milk (titrated acidity) and the well-being of the farm (diseases), since milk from sick cows has increased bacterial contents and somatic cells. In addition, bacterial contamination can be used to assess the technology of milk production and compliance with veterinary and sanitary rules for milking animals [15]. Somatic cell counts (SCCs) are an important parameter in udder health, since somatic cells are involved in protecting the mammary glands from infection as part of the animal’s immune system. SCC in milk is affected by many factors, including species, level of milk production, lactation stage, management methods, and a variety of individual and environmental factors [16].

The aim of this study was to determine the effect of animal breed, season, and milk production on the physicochemical characteristics of milk and predicting the effect of these changes on the composition and quality of milk products.

## Materials and Methods

### Ethical approval and Informed consent

No ethical approval was required; however, during the collection of samples, verbal permission was taken from the farm owners and farm workers.

### Study area and period

Milk was obtained from LLP “Kirova” of Pavlodar region (Simmental breed) and JSC “Astana-Onim” of Akmola region (Holstein breed) of the Republic of Kazakhstan. Samples were obtained from June 2017 to May 2018. The physicochemical characteristics of milk were assessed at the laboratory of LLC Kazakh Research Institute of Livestock Breeding and Fodder Production using a high-performance, fully-automatic milk analyzer (MilkoScan FT+; Fossomatic FT+).

### Animals

The composition and process properties of milk were studied using 80 milk samples from 40 Simmental and 40 Holstein cows. The cows at the dairy farm were kept loose on a natural pasture. Grazing of farm animals was carried out on fenced pastures. They had water *ad libitum*.

### Experimental design

The samples were prepared and FA compositions were determined in accordance with the State Standard 32915-2014 [17]. FA was determined using a gas chromatograph GC Shimadzu-2010 Plus with a flame ionization detector and a capillary column Agilent J&W Columns GP-Sii 88 for FAME with the dimensions of 100 m×0.25 mm× 0.2 mL. Nitrogen, hydrogen, and air were supplied to the detector from the gas flow regulator; the maximum detector temperature was 260°C with temperature settings at 100°C for 5 min, up to 210°C for 8 min at the speed of 4°C/min, up to 240°C for 25 min at the speed of 10°C/min; the volume of the injected sample was 1 μL. The splitting flow was 1/40.

### Statistical analysis

The obtained data were processed through variation statistics using the Statistica software package for Windows version 6.0 (Stat Soft Inc., USA).

## Results

It should be noted that the variation of the main breeding characteristics generally obeys the laws of normal distribution (p<0.05): The fat content in milk (in Holsteins *Χ*^2^*emp* =0.138 at *Χ*^2^*theor* 0.95 =3.84; in Simmentals *Χ*^2^*emp* =2.711 at *Χ*^2^*theor* 0.95 =5.99), the protein content in milk (in Holsteins *Χ*^2^*emp* =0.773 in Simmentals *Χ*^2^*emp* =1.196), linoleic FA (in Holsteins *Χ*^2^*emp* =0.146 in Simmentals *Χ*^2^*emp* =1.889), and so on. According to some signs (for example, a_linolenic (omega 3, somatic cell content), an asymmetric distribution is observed.

The obtained physicochemical and microbiological milk quality indicators and their statistical characteristics for a period of 305 lactation days are presented in Table-1. On average, the Simmental milk contained 3.39% protein and 4.02% fat. The Holstein milk contained an average 3.22% protein and 3.72% fat. The differences in fat content between the two breeds were statistically significant, with Simmental milk having higher fat contents in summer and winter (p<0.001 and p<0.05, respectively), while the other periods of the year were random (p>0.05).

**Table 1:** Physicochemical and microbiological indicators of milk by season.

Biometric indicators	Mass fraction of fat, %	Mass fraction of protein, %	The content of somatic cells, thousand per 1 cm^3^	Acidity, ^0^T	Density, g/?m^3^
Spring					
Holstein (n*=*40)					
x̄±m_x̄_	3.70±0.04	3.18±0.05	260.11±45.40	16.80±16.8	1027.00±0.15
*σ*	0.26	0.30	287.17	0.79	0.96
*Cv*	7.13	9.58	110.40	4.71	0.09
Simmental (n*=*40)					
x̄±m_x̄_	3.94±0.06	3.32±0.07	330.57±78.19	16.80±0.11	1028.00±0.19
*σ*	0.37	0.43	494.54	0.69	1.22
*Cv*	9.27	12.96	149.60	4.10	0.12
d_x̄_1__-_x̄_2__	0.24	0.14	70.46	0	1
*t_d_/p*	3.33/<0.01	1.63/>0.05	0.78/>0.05	0.00/>0.05	4.13/<0.001
**Summer**					
Holstein (n*=*0)					
x̄±m_x̄_	3.73±0.05	3.17±0.05	167.96±24.06	18.00±0.08	1028.00±0.21
*σ*	0.30	0.35	152.15	0.51	1.32
*Cv*	7.97	10.89	90.59	2.81	0.13
Simmental (n*=*40)					
x̄±m_x̄_	3.99±0.04	3.45±0.03	384.24±72.81	17.00±0.13	1028.00±0.18
*σ*	0.27	0.20	460.48	0.82	1.15
*Cv*	6.82	5.75	119.84	4.80	0.11
d_x̄_1__-_x̄_2__	0.26	0.28	-216.28	1	0
*t_d_/p*	4.06/<0.001	4.80/<0.001	2.82/<0.05	6.55/<0.001	0.00/>0.05
**Autumn**					
Holstein (n*=*40)					
x̄±m_x̄_	3.80±0.04	3.30±0.06	282.77±31.36	17.00±0.15	1027.00±0.14
*σ*	0.26	0.38	198.31	0.92	0.88
*Cv*	6.96	11.47	70.13	5.40	0.09
Simmental (n*=*40)					
x̄±m_x̄_	4.04±0.04	3.39±0.02	405.81±41.78	17.00±0.12	1029.00±0.16
*σ*	0.24	0.14	264.21	0.78	0.99
*Cv*	5.89	4.11	65.11	4.61	0.10
d_x̄_1__-_x̄_2__	0.24	0.09	123.04	0	2
*t_d_/p*	4.24/<0.001	1.42/>0.05	2.36/<0.05	0.00/>0.05	9.41/<0.001
**Winter**					
Holstein (n*=*40)					
x̄±m_x̄_	3.64±0.06	3.22±0.05	343.92±96.86	16.90±0.13	1027.00±0.14
*σ*	0.37	0.32	612.61	0.84	0.88
*Cv*	10.03	9.81	178.13	4.98	0.09
Simmental (n*=*40)					
x̄±m_x̄_	4.09±0.08	3.41±0.07	410.98±90.74	16.70±0.10	1027.00±0.16
*σ*	0.50	0.45	573.87	0.65	0.99
*Cv*	12.25	13.10	139.64	3.88	0.10
d_x̄_1__-_x̄_2__	0.45	0.19	67.06	0.2	0
*t_d_/p*	4.50/<0.001	2.21/<0.05	0.51/>0.05	1.22/>0.05	0.00/>0.05

A higher content of somatic cells in the Simmental milk was measured during all periods of lactation: 330.57 × 10^3^ cells/cm^3^; 384.24 × 10^3^ cells/cm^3^; 405.81 × 10^3^ cells/cm^3^; and 410.98 × 10^3^ cells/cm^3^. According to European standards, cow milk is allowed to have 250 thousand somatic cells per 1 cm^3^. According to the Technical Regulations of the Customs Union 033/2013 “On the Safety of Milk and Dairy Products,” cow milk is allowed to have 750 thousand somatic cells per 1 cm^3^. If the number of somatic cells exceeds this value, then the milk is considered to be of insufficient quality for use in high-quality dairy products due to the milk’s corresponding low casein, milk sugar, calcium, magnesium, and phosphorus contents [18]. From the data presented in Table-1, we can notice very high lability of the somatic cell content in all seasons of the year: The coefficient of variability varies from 65.11% (Simmental) to 178.13% (Holstein). A relatively low level was observed in the autumn period.

The seasonal variability of the other physical and chemical parameters was within the normal range. Milk acidity was higher in the Holstein cows (p<0.05). It should be noted that other seasons and years in both breeds did not display significant differences. The mass fraction of dry skimmed milk substances in Simmental cows in the spring, summer, autumn, and winter periods was 8.57%; 8.62%; 8.7%; and 8.71%, and 8.56%; 8.88%; 8.64%; and 8.48% in Holstein cows, respectively.

The results of the two-factor analysis of variance are given in Table-2, which shows the proportion of influence that the studied factors (breed and season) had separately, and their combined effect on the physicochemical and microbiological milk parameters. Breed had a highly reliable influence on all studied indicators, except for milk acidity, but season had only a random effect (except for milk density). The ratio of factorial variance to total variances was from 7.04 (somatic cell content) to 30.69% (milk density),in all cases, F*_emp_>*F*_st_* (for somatic cell content: p<0.01; for the rest: p<0.001).

**Table 2:** Influence of the breed and season on the physicochemical and microbiological parameters of milk.

A (breed)		B (season of the year)	AB (the joint effect of)	*x*	*z*	*y*
Fat content in milk						
*D*	105	8.71	13.46	126.8	527.4	654.2
*ƞ*	0.16	0.01	0.02	0.19	0.81	1.00
*σ^2^*	104.65	2.90	4.49	18.12	1.69	2.05
*F_emp_*	61.91***	1.72	2.65*	10.72***	–	–
Protein content in milk						
*D*	66.613	10.26	11.11	88.0	882.9	970.9
*ƞ*	0.07	0.01	0.01	0.09	0.91	1.00
*σ^2^*	66.61	3.42	3.70	12.57	2.83	3.04
*F_emp_*	23.54***	1.21	1.31	4.44***	–	–
The content of somatic cells, thousand per 1 cm^3^					
*D*	8.72	2.99	6.09	17.8	235.1	252.9
*ƞ*	0.03	0.01	0.02	0.07	0.93	1.00
σ^2^	8.7	1.00	2.03	2.54	0.76	0.80
F_emp_	11.5***	1.31	2.67*	3.34**	–	–
Density, *г/CM^3^*						
*D*	45.00	55.00	55.00	155.0	350.0	505.0
*ƞ*	0.09	0.11	0.11	0.31	0.69	1.00
*σ^2^*	45.0	18.33	18.33	22.14	1.12	1.58
F_emp_	40.1***	16.34***	16.34***	19.74***	–	–
Acidity, *^0^Т*					
*D*	0.15	3.01	0.66	3.8	182.2	186.0
*ƞ*	0.001	0.02	0.004	0.02	0.98	1.00
*σ^2^*	0.2	1.00	0.22	0.55	0.58	0.58
*F_emp_*	0.3	1.72	0.38	0.94	–	–

*** –р<0.001;

** –р<0.01;

* –р<0.05

The seasonal dynamics of mass fractions of the 16 main FA in the milk fat is given in Tables-3 and 4. Some FA were not included in the list, since their relative peak areas exceeded 0.1%. As expected, Saturated FA (SFA) represented the most common class followed by monounsaturated FA (MUFA) and polyunsaturated FA (PUFA). The most common FA in both groups were C16:0, which is the sum of the isomers C18:1, C18:0, and C14:0. No statistical difference in the concentrations of these compounds was observed between the two studied breeds. In addition, short- and medium-chain SFA, such as C4:0, C6:0, C8:0, C10:0, and C12:0, were present at the expected levels. In the spring and summer, relatively high butyric acid C4:0 content was noted in the milk of both breeds, which is characteristic of milk fat, and is involved in the flavor creation of dairy products.

**Table 3:** Fatty acid composition of milk in the spring-summer period.

Fatty acid name	X̄	m_X̄_	*Cv*	X̄	m_X̄_	*Cv*	Comparison of breeds		
		
	Holstein			Simmental			*d*	*t_d_*	*p*
Spring									
C4:0	3.29	0.08	15.12	3.49	0.07	12.76	0.20	1.88	>0.05
C6:0	1.78	0.05	16.56	1.56	0.01	5.11	0.22	4.18	<0.001
C8:0	1.16	0.02	9.48	1.09	0.01	5.74	0.07	3.20	<0.01
C10:0	3.05	0.10	20.34	2.21	0.04	11.68	0.84	7.77	<0.001
C10:1	0.29	0.01	20.52	0.23	0.01	13.71	0.06	5.03	<0.001
C12:0	3.11	0.09	18.48	2.71	0.08	18.32	0.40	3.33	<0.01
C14:0	9.99	0.20	12.69	10.48	0.17	10.13	0.49	1.89	>0.05
C14:1*	1.23	0.04	22.57	1.03	0.03	18.48	0.20	3.91	<0.001
C16:0	29.29	0.31	6.65	30.14	0.13	2.67	0.85	2.54	>0.05
C16:1*	2.28	0.03	8.34	1.70	0.03	9.90	0.58	14.50	<0.001
C18:0	9.84	0.21	13.48	8.26	0.04	2.98	1.58	7.42	<0.001
C18:1*	25.14	0.31	7.88	29.25	0.22	4.76	4.11	10.82	<0.001
C18:2*	3.84	0.14	22.41	3.04	0.07	14.98	0.80	5.08	<0.001
C18:3*	1.29	0.05	22.39	0.42	0.01	18.95	0.87	16.84	<0.001
C20:0	0.20	0.01	30.00	0.13	0.00	16.09	0.07	6.38	<0.001
C22:0	0.09	0.02	128.36	0.06	0.00	24.11	0.03	1.41	>0.05
Other	4.13	0.03	4.33	4.18	0.03	4.36	0.05	1.14	>0.05
Summer									
C4:0	3.66	0.06	10.10	3.77	0.06	9.79	0.11	1.30	>0.05
C6:0	1.88	0.06	18.71	1.83	0.05	16.45	0.05	0.64	>0.05
C8:0	1.48	0.05	20.65	1.21	0.03	17.39	0.27	4.63	< 0.001
C10:0	2.95	0.07	14.94	2.71	0.09	21.56	0.24	2.10	>0.05
C10:1	0.34	0.01	20.30	0.27	0.01	17.46	0.07	4.95	<0.001
C12:0	3.11	0.08	16.03	2.39	0.06	16.60	0.72	7.20	<0.001
C14:0	11.29	0.20	11.06	8.97	0.10	6.85	2.32	10.38	<0.001
C14:1*	1.18	0.03	16.14	1.24	0.04	21.19	0.06	1.20	>0.05
C16:0	28.06	0.27	6.14	27.70	0.24	5.57	0.36	1.00	>0.05
C16:1*	1.99	0.05	15.50	1.96	0.05	14.98	0.03	0.42	>0.05
C18:0	11.32	0.21	11.59	11.19	0.17	9.52	0.13	0.48	>0.05
C18:1*	23.61	0.29	7.87	28.48	0.24	5.39	4.87	12.94	< 0.001
C18:2*	3.84	0.12	19.97	2.49	0.04	10.14	1.35	10.67	< 0.001
C18:3*	0.90	0.04	29.22	1.06	0.05	29.28	0.16	2.50	>0.05
C20:0	0.15	0.01	21.70	0.14	0.01	29.48	0.01	0.71	>0.05
C22:0	0.10	0.00	9.43	0.07	0.003	26.75	0.03	10.00	<0.001
Other	4.16	0.02	3.13	4.52	0.07	9.54	0.36	4.94	<0.001

* The calculation is based on the sum of the isomers. by *v*=40+40–2=78 *t_st_*=1.98 (p=0.05)–2.63 (p=0.01)–3.39 (p=0.001)

**Table 4:** Fatty acid composition of milk in the autumn-winter period.

Fatty acid name	X̄	m_X̄_	*Cv*	X̄	m_X̄_	*Cv*	Comparison of breeds		
		
	Holstein			Simmental			*d*	*t_d_*	*p*
Autumn									
C4:0	3.27	0.07	14.28	3.32	0.09	16.52	0.05	0.44	>0.05
C6:0	1.58	0.01	4.27	2.47	0.06	15.74	0.89	14.63	<0.001
C8:0	1.23	0.02	12.18	1.43	0.04	19.08	0.20	4.47	<0.001
C10:0	2.08	0.01	2.62	3.03	0.09	18.50	0.95	10.49	<0.001
C10:1	0.26	0.01	16.06	0.31	0.01	19.61	0.05	3.54	<0.001
C12:0	2.71	0.06	15.12	3.75	0.07	12.05	1.04	11.28	<0.001
C14:0	10.03	0.16	9.82	11.45	0.19	10.49	1.42	5.72	<0.001
C14:1*	1.29	0.03	13.30	1.37	0.02	9.42	0.08	2.22	>0.05
C16:0	28.27	0.21	4.75	30.79	0.28	5.66	2.52	7.20	<0.001
C16:1*	2.09	0.05	13.89	2.38	0.01	1.40	0.29	5.69	<0.001
C18:0	9.02	0.10	7.31	9.01	0.12	8.43	0.01	0.06	>0.05
C18:1*	29.25	0.23	5.04	22.69	0.25	7.07	6.56	19.31	<0.001
C18:2*	3.60	0.13	23.62	2.46	0.05	12.91	1.14	8.18	<0.001
C18:3*	1.01	0.05	33.37	0.67	0.04	36.17	0.34	5.31	<0.001
C20:0	0.13	0.004	20.58	0.12	0.003	16.92	0.01	2.00	>0.05
C22:0	0.07	0.002	20.11	0.07	0.003	27.84	0.00	0.00	>0.05
Other	4.09	0.05	7.96	4.67	0.08	10.98	-0.58	6.15	<0.001
Winter									
C4:0	2.83	0.06	14.37	3.20	0.08	15.79	0.37	3.70	<0.001
C6:0	1.78	0.04	12.63	1.59	0.02	7.61	0.19	4.25	<0.001
C8:0	1.27	0.03	17.28	1.32	0.03	16.73	0.05	1.18	>0.05
C10:0	2.93	0.08	17.79	2.01	0.02	5.06	0.92	11.16	<0.001
C10:1	0.30	0.01	20.73	0.21	0.00	5.91	0.09	8.83	<0.001
C12:0	3.31	0.09	17.72	2.50	0.06	15.73	0.81	7.49	<0.001
C14:0	11.03	0.18	10.21	9.42	0.13	8.62	1.61	7.25	<0.001
C14:1*	1.12	0.03	19.21	1.40	0.01	6.57	0.28	8.85	<0.001
C16:0	27.76	0.35	8.03	28.03	0.26	5.91	0.27	0.62	>0.05
C16:1*	2.08	0.04	11.90	1.55	0.01	3.78	0.53	12.85	<0.001
C18:0	9.94	0.16	9.95	10.82	0.18	10.71	0.88	3.65	<0.001
C18:1*	26.17	0.32	7.73	28.91	0.26	5.68	2.74	6.65	<0.001
C18:2*	2.91	0.10	21.64	3.35	0.11	20.39	0.44	2.96	<0.01
C18:3*	0.80	0.04	34.38	1.17	0.04	22.17	0.37	6.54	<0.001
C20:0	0.22	0.007	21.08	0.23	0.01	20.94	0.01	0.82	>0.05
C22:0	0.09	0.003	19.46	0.10	0.003	22.34	0.01	2.36	>0.05
Other	5.47	0.09	10.76	4.19	0.05	7.75	1.28	12.43	<0.001

* The calculation is based on the sum of the isomers. by v=40+40–2=78 t_st_=1.98 (p=0.05)–2.63 (p=0.01)–3.39 (p=0.001)

Among MUFA, the C18:1 isomers were the main components. In general, the remaining percentage of monounsaturated FA in the total amount of milk fat in the Simmental breed was 2.96%; 3.47%; 4.06%; and 3.16%; and 3.8%; 3.51%; 3.64%; and 3.5% in the Holstein breed. All of which were significantly higher in the milk from the Holstein cows. PUFA in the Simmental breed was 3.46%; 3.55%; 3.13%; and 4.52% and in the Holstein breed was 5.13%; 4.74%; 4.61%; and 3.71% of the total amount of FA. In both groups of milk, C18:2n-6 was the predominant compound. The percentage of ὠ −3 PUFA was higher in the samples from the Simmental breed (1.06% and 1.17% vs. 0.97% and 0.8%) in the summer and in the winter compared to the Holstein breed; however, the percentage of ὠ −3 PUFA in the spring and autumn periods in the Holstein breed was 1.29% and 1.01% versus 0.42% and 0.67%. The percentage of ὠ −6 PUFA (3.84%; 3.84%; and 3.6% vs. 3.04%; 2.49%; and 2.46%) in the spring, summer, and the autumn was higher in the Holstein breed than the Simmental breed.

A noticeable interbreed difference was observed in the C10:1, C12:0, C16:1, C18:1, and C18:3 FA contents: The first two-three acids were higher in the Holstein cows, but C18:1 was higher in the Simmental cows (except for in the autumn). Interbreed differences in the FA C6:0, C10:0, C14:0, C16:1, and C18:3 were also statistically significant, except for some seasons of the year (mainly the summer). It is noteworthy that the mass fraction of C6:0, C8:0, and C10:0 FA in the spring-summer and winter periods was higher in the Holstein cows, but that the autumn period was higher in the Simmental cows (p<0.001). There were no noticeable interbreed differences in the sum of the isomers C4:0, C16:0, C20:0, and C22:0 (p>0.05). The statistical significance of the interbreed differences of the remaining isomers is shown in Tables-3 and 4.

Very important is the content of such FA as omega-6 and omega-3 in milk. In this regard, for a more detailed judgment of the share of the influence of the breed and seasons of the year, we conducted a two-factor analysis of variance. The results of the analysis are shown in Table-5. These tables also confirm the earlier conclusion that there was a noticeable breed influence (p<0.001) on milk quality.

**Table 5:** Two-factor analysis of variance.

A (breed)		B (season of the year)	AB (the joint effect of)	*x*	*z*	*y*
C18:2						
*D*	154.01	18.36	143.76	316.14	518.55	834.69
*ƞ*	0.18	0.02	0.17	0.38	0.62	1
*σ^2^*	154.01	6.12	47.92	45.16	1.66	2.62
*F_emp_*	92.67	3.68	28.83	27.17	–	–
*p*	< 0.001	< 0.05	< 0.001	< 0.001	–	–
C18:3						
*D*	98.62	49.20	813.90	961.72	972.37	1934.09
*ƞ*	0.05	0.03	0.42	0.50	0.50	1
*σ^2^*	98.62	16.40	271.30	137.39	3.13	6.08
*F_emp_*	31.54	5.25	86.77	43.94	–	–
*p*	<0.001	<0.01	<0.001	<0.001	–	–

## Discussion

A component that is essential in the processing of milk into finished dairy products is milk fat. Fat is the most important quality and economic indicator of cow milk. On average, milk contains 3.7-3.8% fat with a significant variability [19]. The milk in our study corresponded to these averages at 3.7-4.0%.

The number of somatic cells in milk is a relatively poorly studied indicator of safety, although it is clear that a reduced number of somatic cells improve milk quality. Studies have shown that the number of somatic cells in milk changes depending on calving season [20]. The largest number of somatic cells was found in the milk from spring calving cows (237 and 252 × 10^3^cells/cm^3^), which may be due to the extreme weather conditions. The smallest number of somatic cells occurs in summer calving cows (89 and 99 × 10^3^ cells/cm^3^). These results concur with those of our study, with 167.96 thousand somatic cells measured in 1 cm^3^.

The quantitative and qualitative FA composition changes slightly in each season. Gorelik *et al*. [21] found the lowest amount of conjugated acids in winter, and the highest amounts in summer, after which the amount gradually reduced. These changes are likely due to the presence of green fodder rich in easily digestible carbohydrates available to the cows in the summer but not the winter.

Saturated FA (EFAS) dominates milk fat and ranges from 67.11% to 67.89%, while unsaturated FA ranges from 31.8% to 32.16% [22]. Milk and dairy products are the most common source of conjugated linoleic acid. Two FAs linoleic acid (LA, C18:2 n-6) and a-linoleic acid (ALA, C18:3 n-3) are nutritionally important, since they cannot be synthesized by humans. Human cells are included in this group along with other favorable FAs, such as conjugated linoleic acids [23]. In our studies, the content of EFAS ranged from 58.39% to 65.44% depending on the season and breed, and the content of monounsaturated FA ranged from 26.75% to 32.89% depending on season and breed. The PUFA content was highest in Holstein cows in the spring at 5.13%, and the lowest in Simmental cows in the autumn at −3.13%. These data differ from the average literature values for other breeds, which had a high content of palmitic and stearic acids and a low content of polyunsaturated acids in winter, and a reverse trend in summer [24-26]. This discrepancy may be due to changes in the diet during these periods and breed characteristics.

## Conclusion

The physicochemical, microbiological parameters and biological value of milk from Simmental and Holstein cows in different lactation seasons were studied. Notable interbreed differences were found in the physicochemical and microbiological characteristics of milk (except for milk density from the summer period), with the Simmental breed superior to the Holstein breed in regard to the fat content and the number of somatic cells in milk. According to all the studied indicators (except for milk acidity), breed had a highly reliable effect on milk quality, but seasonal differences were random (except for milk density). The ratio of factorial variance to total variances ranged from 7.04 (somatic cell content) to 30.69% (milk density), in all cases F*_emp_>*F*_st_*. Statistically significant interbreed differences in FA (C6:0, C10:0, C14:0, C16:1, and C18:3) were also generally found. It is noteworthy that the mass fraction of caproic, caprylic, and capric FA in the spring-summer and winter periods was higher in the milk of Holstein cows and in the Simmental cows in the autumn. There were no noticeable interbreed differences in the sum of the isomers C4:0, C16:0, C20:0, and C22:0. In order to increase the concentration of the fat phase of milk and increase its biological value, it is necessary to optimize the diets of highly productive cows for all nutrients.

## Authors’ Contributions

AZK: Acquisition of data, analysis and interpretation of data, drafting the article, conception and design, revising it critically, and final approval. VSZ: Acquisition of data, analysis and interpretation of data, drafting the article, conception and design, revising it critically, and final approval. LAM: Acquisition of data, analysis and interpretation of data, drafting the article, conception and design, revising it critically, and final approval. ATK: Acquisition of data, analysis and interpretation of data, drafting the article, conception and design, revising it critically, and final approval. NZK: Acquisition of data, analysis and interpretation of data, drafting the article, conception and design, revising it critically, and final approval. KMM: Acquisition of data, analysis and interpretation of data, drafting the article, conception and design, and revising it critically. All authors have read and approved the final manuscript.
